# Molecular Dynamics Simulation of the Implantation of *b*-Oriented ZSM-5 Film Modified α-Quartz Substrate Surface With Different Modifiers

**DOI:** 10.3389/fchem.2019.00746

**Published:** 2019-11-06

**Authors:** Jiaxin Wu, Xianliang Meng, Ruizhi Chu, Shi Yu, Yongzhou Wan, Chengcheng Song, Guifeng Zhang, Tong Zhao

**Affiliations:** ^1^School of Chemical Engineering and Technology, China University of Mining and Technology, Xuzhou, China; ^2^Key Laboratory of Coal Processing and Efficient Utilization of Ministry of Ministry of Education, Xuzhou, China

**Keywords:** *b*-oriented ZSM-5 film, modified α-quartz, implantation, molecular dynamics simulation, MTA reaction

## Abstract

In this study, we investigated the structure–absorption relationship of common surface modifiers of chitosan (CTS), polyvinyl acetate (PVA), and titanium dioxide (TiO_2_) with α-quartz surface using molecular dynamics simulation. And the orientations and combinations derived from structures between modified α-quartz and ZSM-5 crystallites were also investigated. The results show that PVA is a non-linear organic macromolecule with a large amount of hydroxyl groups on its surface, which easily adhere to the surface of the substrate and agglomerate. CTS is a straight-chain structure containing a large number of hydroxyl and amino groups, which easily accumulate and spread on the surface of the substrate. TiO_2_ not only forms hydrogen bonds and complexes with the substrate but also interacts with each other to form a dense modifier layer. We observed that a large number of stable Ti–O–Si chemical bonds formed between the modified layer of inorganic small-molecule TiO_2_ and the surface of α-quartz, which compacted and stabilized the attached ZSM-5 film. Moreover, the orientation angle of the ZSM-5 nanocrystalline nucleus on the modified α-quartz was computed, which confirmed that the *b*-axis orientation of the ZSM-5 nanocrystalline nucleus was the highest on the surface of the substrate modified by TiO_2_. We discussed the influence of the modified temperature of modifiers in the constructed materials, and we have observed the adsorption state differences of TiO_2_ at different modified temperatures. We also discussed the catalytic properties of the materials prepared by the corresponding methods in conversion of methanol-to-aromatics (MTA) reaction. These results agree with our previous experimental data. By employing molecular dynamics simulation, we have obtained more precise conclusive information of the *b*-oriented growth of ZSM-5 crystallites, which highly depends on the surface modifiers.

**Graphical Abstract:**
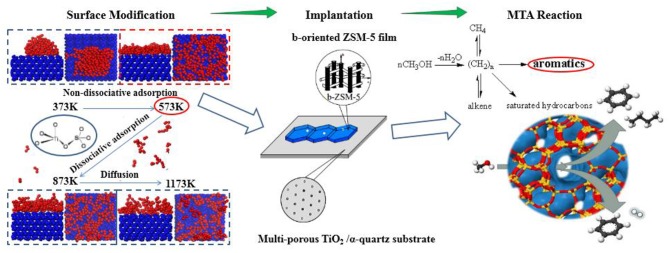
Determination and utilization of *b*-oriented ZSM-5 film on TiO_2_/α-quartz substrate.

## Highlights

- Model building and optimization of *b*-oriented ZSM-5/modifiers/substrate film by molecular dynamics simulation.
- Effect of orientations and combinations of implantation of *b*-oriented ZSM-5 film modified α-quartz substrate surface with different modifiers.
- *b*-Oriented ZSM-5/TiO_2_/substrate catalyst significantly improved methanol conversion, aromatic hydrocarbon yield, and lifetime.

## Introduction

ZSM-5 belongs to the MFI topology zeolite and has a three-dimensional pore structure. The growth in the vertical direction is the *b*-axis ZSM-5 (*b*-ZSM-5) zeolite. The straight-channel structure of the *b*-axis (0 1 0) of ZSM-5 enables the molecules to rapidly diffuse in a one-dimensional straight channel, shortening the molecular diffusion path, thereby reducing the possibility of deep side reactions of the product and inhibiting the formation of carbon deposits. ZSM-5 molecular sieves have high stability, have high specific surface area and selective catalytic function, and is often used in methanol-to-aromatics (MTA), methanol-to-olefins (MTO), and methanol-to-hydrocarbon (MTH) reactions. Since Feng and Bein (1994) realized the control of molecular sieve crystal orientation for the first time, MFI molecular sieve membranes with *a*-axis orientation, *b*-axis orientation, *c*-axis orientation and composite orientation have been prepared on various carriers by controlling the interaction between carrier and molecular sieve crystal.

Since researchers proposed that the structure and physicochemical properties of the substrate surface would affect the nucleation and growth of molecular sieves (Koegler et al., 1997), they thought that the synthetic liquid would first form a gel layer on the surface of the silicon-rich silicon hydroxyl, and the supersaturation was larger at the interface between the gel layer and the liquid main interface. The nuclei form at the interface between the two phases, and then the crystals grow on the surface of the *b*-axis orientation carrier through hydrogen bonding force to form *b*-axis orientation molecular sieve films. The high orientation molecular sieve materials were obtained by modifying the surface of the substrate with modifiers. Lee et al. (2003) prepared three orientations of pure silicon MFI (silicalite-1) molecular sieve crystal *a*-, *b*-, and *c*-axes by pre-coating a highly ordered polyurethane film modifier on the glass surface. When the surface groups are randomly oriented polyurethane films, only a randomly oriented silicalite-1 film can be obtained. Obviously, the functional groups and their ordered alignment on the surface of polyimide films are the key to achieving directional guidance and to obtaining ordered alignment of molecular sieve arrays. Lang et al. (2009) synthesized silicalite-1 molecular sieve membrane with *b*-axis orientation on the surface of chitosan (CTS)-modified porous silica–zirconium composite membrane carrier (average pore diameter is 8 nm). A silicalite-1 molecular sieve membrane with continuous coverage and *b*-axis orientation formed on the surface of the silica–zirconium composite interlayer modified by soluble carboxymethyl CTS (CMCTS). Wang and Yan (2001) found that the material and roughness of the substrate had a great influence on the orientation of molecular sieve membranes. Silicalite-1 molecular sieve membranes with *b*-axis orientation could be synthesized on polished smooth stainless steel substrates. In summary, the material control of ZSM-5 molecular sieve membranes with high preference in orientation mainly focused on the surface modification of the substrate. The modifiers used for substrate modification are mainly organic and inorganic modifiers. The common organic modifiers are polyvinyl acetate (PVA), CTS, etc. (Wang et al., 2010; Wei et al., 2017), and inorganic modifiers are TiO_2_, SiO_2_, etc. (Liu et al., 2011; Cheng et al., 2014). The surface of PVA has a large number of hydroxyl functional groups, which easily adhere to the surface of quartz substrate to form active groups and easily form hydrogen bonds with ZSM-5 nanocrystalline nuclei to promote binding. There are a large number of hydroxyl, amino, and ether bonds on the surface of CTS, which easily form hydrogen bonds with ZSM-5 nanocrystalline nuclei. As a small molecule, TiO_2_ can form a smooth dressing layer on the surface of the substrate, and various crystal structures formed at different temperatures, which enhances the *b*-axis orientation growth of the seed.

At present, the synthesis of *b*-ZSM-5 composites faces difficulties. Experimental conditions are hard to control accurately for the traditional method. The interface effects of *b*-ZSM-5 molecular sieves, modifier, and substrate cannot be studied in detail. Therefore, the design of new ZSM-5 molecular sieve membrane materials cannot be guided. Molecular dynamics (MD) simulation overcomes the shortcomings of experiments and can simulate the system at atomistic scale. In recent years, many researchers have successfully used MD calculation to explain the interface effect between systems. Yan et al. (2016) simulated the interactions between PVA and poly(methyl methacrylate) (PMMA) on α-quartz surface by MD. Liu et al. (2015) studied the adsorption of DDA, ether amine, and AC1210 on α-quartz at different acidity and alkalinity by a universal force field (UFF) in MD simulations. MD simulation has been used in the design of composite molecular sieve materials. It has been reported that the epitaxial growth evidence of ZnO, Fe–B, and carbon nanotube (Fang et al., 2006; Tarabaev and Esin, 2007; Sharma et al., 2015) can be well-explained by MD simulation, and the results are basically consistent with the experiment. Brinkmann et al. (2011) has focused on the interfacial effects between ZSM-5 and quartz with different gap sizes by MD simulation.

In this work, we attempt to design a model system to investigate the thermodynamic behaviors of *b*-ZSM-5 implanted on the surface of α-quartz substrate with organic and inorganic modifiers (PVA, CTS, and TiO_2_) at a molecular level. Equilibrium all-atom MD simulations were used to study the whole system in vacuum. Mean square displacement (MSD), diffusion coefficient, relative concentration distribution, and hydrogen bonds were analyzed to investigate the preplantation mechanisms of *b*-ZSM-5 on the surface of modified α-quartz, and the orientation angle (Zeng Y. Q. et al., 2015) between *b*-ZSM-5 and modified substrate is also calculated to explain the orientation degree between *b*-ZSM-5 and the substrate surface. Our long-term goal is to fully understand the binding between *b*-ZSM-5, modifiers, and α-quartz and how the reactive functional groups affect the binding processes, which is vital for the preparation of ZSM-5 catalyst materials designed with high *b*-axis orientation.

## Materials and Methods

### Material Synthesis

#### Preparation of Modified α-Quartz

1. Substrate surface modification

The α-quartz sieve from the coal mine of Zhouyuanshan was crushed and then passed through a 200-mesh sieve. The α-quartz powder was calcined in a muffle furnace at 850°C for 2 h and then compression-molded to obtain the raw substrate, before acid treatment that consisted of 15% HCl and H_2_SO_4_ with a volume ratio of 1:1. The raw substrate was calcined in a muffle furnace at 1,123 K for 1 h. The side of the α-quartz substrate was polished with 1000#, 1500#, and 2000# sandpaper until the surface is smooth, and the α-quartz substrate was placed for ultrasonic cleaning for 10 min.

An aqueous solution of 4% PVA and an acetic acid solution of 0.5% CTS were used to modify the substrate for several times by the dip-coating method, and then the dip-coating substrate was dried at 333 K for 12 h. Ethanol sol at 10% TiO_2_ was applied to modify the substrate at 373 K by the sol-gel method, and the modified substrate was dried for 12 h. After TiO_2_ dip-coating, the substrate was calcined at 573 K.

(2) *b*-ZSM-5 seed layer loading on the modified substrate

A certain amount of tetrapropylammonium hydroxide (TPAOH) and ionized water was weighed with an Erlenmeyer with stirring, and tetraethyl orthosilicate (TEOS) was slowly added to the mixture solution and aging for 24 h. The precursor was crystallized at a temperature of 450 K for 80 min, before the growth of the crystal was stopped with quenching treatment. The material prepared was calcined in a tube furnace with air atmosphere for 2 h at 823 K, and the temperature rise-and-fall rate was 0.5 K/min.

#### Material Characterization

1. Powder X-ray diffraction (XRD) patterns were recorded with a D8 ADVANCE instrument equipped with a graphite monochromator operating at 40 kV and 150 mA using Cu Kα radiation (λ = 0.15 mm). The powder diffractograms were recorded at 0.02° intervals from 5° to 50° with a scanning rate of 2°/min.
2. The morphology and size of the sample were tested by Hitachi's Su8010 scanning electron microscope (SEM), including the morphology, crystal morphology, and size of the substrate surface. To avoid discharge, the sample was subjected to gold spray treatment before the test.
3. Bruker's VERTEX 80v model Fourier transform infrared spectroscopy (FTIR) was used to detect the condition of the organic modifier-modified α-quartz substrate; because it was organically modified on the surface of the substrate, the total reflection infrared spectroscopy attenuated total reflectance (ATR) test was used with a wavelength range from 600 to 4,500 cm^−1^.

### MD Simulation

#### Molecule Models

Potential parameters were taken from Universal, which is parameterized by Johnson et al. (2006). All MD simulations were performed by the program Material Studio (Accelrys). The force field used was of importance in MD simulation. The UFF is an all-atom force field that has parameterizations for every atom of the periodic table with an atomic number lower than 103. The van der Waals interactions are described by the Lennard–Jones potential. Electrostatic interactions are described by atomic monopoles and a screened (distance-dependent) Coulombic term. This force field enables accurate and simultaneous prediction of structural, conformational, and thermophysical properties for silicate compound (Rappe et al., 1992; Froudakis, 2001; Coupry et al., 2017). So UFF is applied to describe the interface and atomic interaction in our simulation. The charge equilibration (QEQ) method is utilized to calculate the charge distributions based on geometry and connectivity throughout the course of the simulation. The ZSM-5 model is derived from the all-silicon MFI molecular sieve that comes from Material Studio (Accelrys). ZSM-5 is designed as a cluster model (Dahl and Kolboe, 1994), retaining the primitive skeleton structure. The suspended silicon atoms are saturated with the hydroxyl group, and the suspended oxygen atoms are saturated with hydrogen atoms. The (1 0 0) crystallographic face of α-quartz was used to model the solid substrate. To obtain a chemically realistic surface, all the non-bridging oxygen atoms were saturated by H atom.

**Figure 1:**
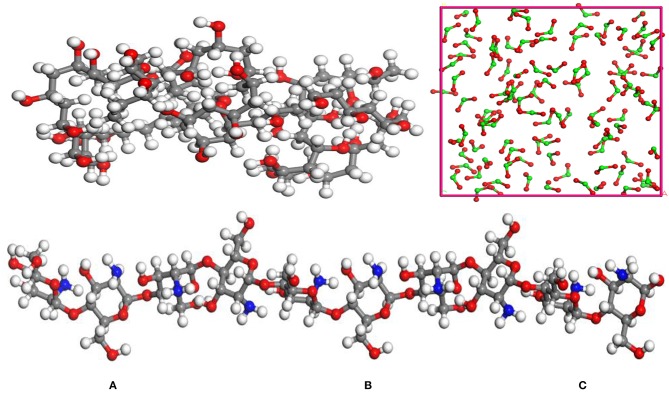
Molecular model of the modifiers: **(A)** polyvinyl acetate (PVA); **(B)** chitosan (CTS); **(C)** TiO_2_. (Gray, red, white, and green represent C, O, H, and Ti, respectively).

TiO_2_, PVA, and CTS were modeled by Material Studio software (Accelrys). In order to make the simulation conditions closer to the molecular weight and agglomeration degree of PVA and CTS in the experiment, the agglomeration degrees of PVA and CTS were set to 40 and 10, respectively, and the repeating unit of the TiO_2_ molecule was set to 100, which is shown in Figure 1.

Periodic models of PVA, CTS, and TiO_2_ were constructed at Amorphous Cell module in Material Studio 8.0. In order to increase the modification degree and improve the flatness of the substrate surface, the periodic model consisting of PVA and CTS with one to five repeat units was built. The PVA-and-CTS molecular model was then added over the α-quartz surface, and there is a distance of 3 Å maintained between the substrate and the modifiers. In addition, in order to eliminate the effects of the upper structure on the model, a vacuum layer of 10 nm is placed above the model. First, 100 TiO_2_ molecules were placed on the substrate surface and at a distance of 3 Å was kept between TiO_2_ and the substrate. Preplanting of ZSM-5 on the modified substrate surface is carried out by placing the *b*-ZSM-5 on the surface of the modified substrate with a distance of 3 Å between the ZSM-5 and modified substrate.

#### Simulation Details

MD simulation for PVA and CTS/substrate: The structure optimization was carried out in the Forcite module to minimize the energy of the system, in which the UFF and the cutoff radius of 12.5 Å were used. MD simulation was carried out in Material Studio 8.0 as follows: at 333 K, with the NVT ensemble and a step size of 1 fs and simulation time of 500 ps using the UFF under the periodic boundary condition.

MD simulation for TiO_2_/substrate: The adsorption state of TiO_2_ on the substrate surface at different temperatures is considered. First, 100 TiO_2_ molecular repeat units were loaded on the surface, and a distance of 3 Å was kept between the surface and TiO_2_. After the structure is optimized to minimize the energy of the system, the MD simulations were performed at 373, 573, 873, and 1,173 K under the NVT ensemble, with a time step size of 1 fs and simulation time of 1,000 ps using the UFF.

MD simulation for ZSM-5/modifier/substrate: After the structure optimization using the Forcite module to minimize the energy of the system, MD simulations were then performed under an NVT ensemble at 450 K, with a step size of 1 fs and simulation time of 1,000 ps using the UFF.

## Results and Discussion

### *b*-ZSM-5/PVA/Substrate MD Simulation

#### Adsorption of PVA on Substrate Surface

**Figure 2:**
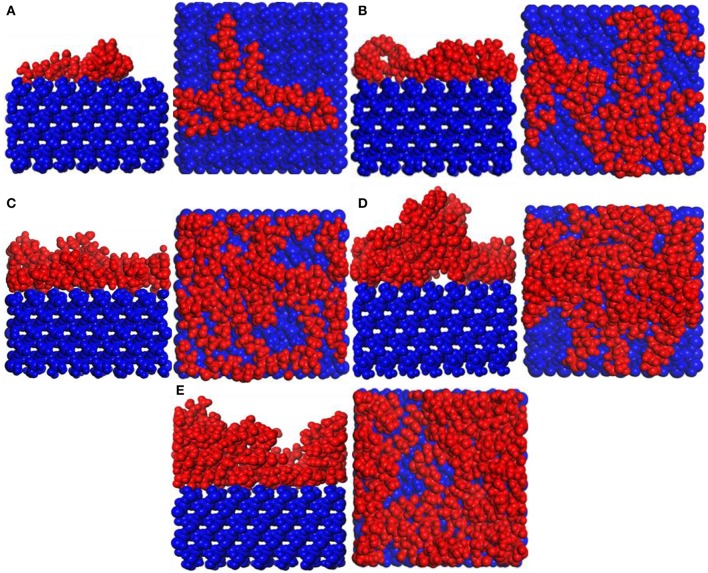
Snapshot diagrams of polyvinyl acetate (PVA)/α-quartz under molecular dynamics simulation: **(A–E)** the repeat units for 1–5.

Figure 2 shows the snapshots of the PVA/α-quartz system. PVA spontaneously adheres to the surface of the substrate because of hydrogen bonding. The PVA molecular configuration is a non-linear structure, so agglomeration occurs on the surface of the substrate (Yan et al., 2016). It can be seen that as the PVA repeat unit increases, the agglomeration of PVA becomes more and more obvious, especially when the number of PVA is equal to 4–5; the irregularity is formed on the surface of the substrate.

**Figure 3:**
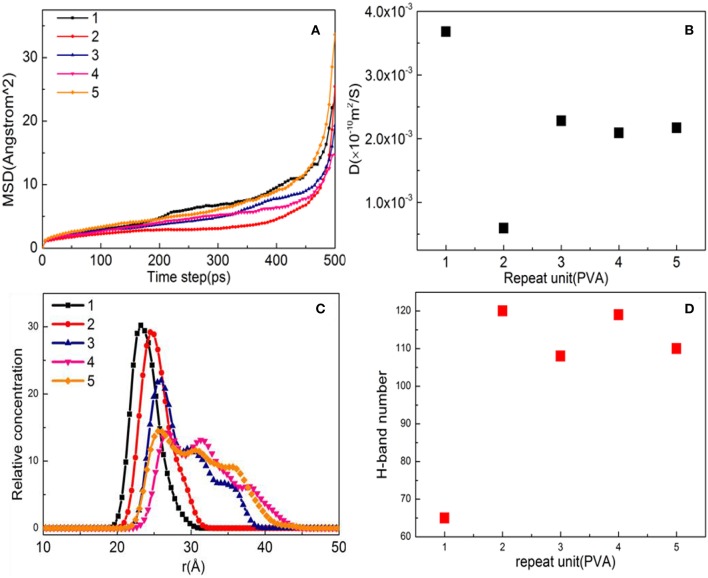
Property analysis of polyvinyl acetate (PVA)/α-quartz substrate after equilibrium of molecular dynamics simulation: **(A)** mean square displacement (MSD); **(B)** diffusion coefficient; **(C)** hydrogen bond number; and **(D)** relative concentration profile.

The higher the value of the MSD and *D*, the stronger is the mobility of the PVA on the surface of the substrate, indirectly indicating the weaker interaction between the surface of the substrate and the PVA (Dai et al., 2017). MSD and *D* (diffusion coefficient) are shown in Figures 3A,B. The MSD curve and the *D* value at different PVA numbers are relatively small, indicating a strong interaction between the PVA and the substrate surface. The order of *D* values of different PVA molecules is 1 > 4 ≈ 5 ≈ 3 > 2, indicating that PVA has the strongest interaction ability when the number is equal to 2. The relative concentration distribution of PVA on the surface of the substrate is shown in Figure 3C. When the number of molecules is 1 and 2, the peak value and the peak shape remain the same and show a single peak, indicating that a single modified layer with a thickness of 10 Å is formed. However, when the number of molecules exceeds 3, small peaks are formed, indicating that a single modified layer was destroyed and the formed modified layer is not uniform. It can also be seen from Figure 3D that the largest number of hydrogen bonds was formed when the number of PVA is equal to 2. Above all, the substrate is fully modified with the 10-Å thickness as the PVA number is 2.

#### Adsorption of b-ZSM-5 on PVA/Substrate Surface

**Figure 4:**
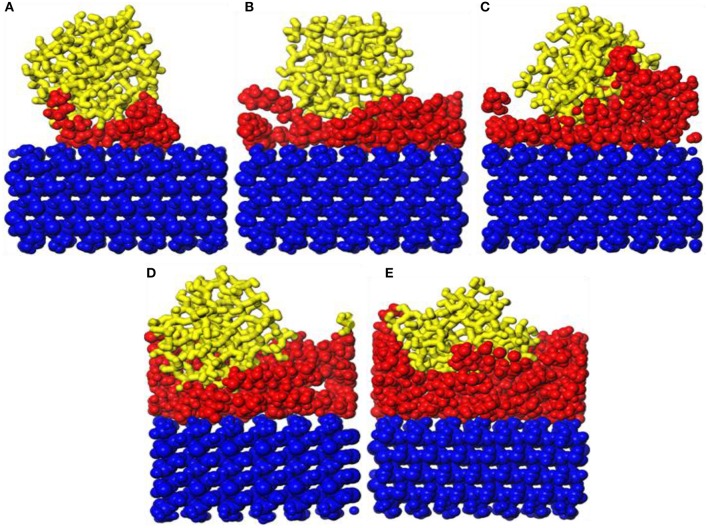
Snapshot diagrams of ZSM-5/polyvinyl acetate (PVA)/α-quartz under molecular dynamics simulation: **(A–E)** the PVA repeat units from 1 to 5.

The snapshot diagrams of the *b*-ZSM-5 on the PVA/substrate surface is displayed in Figure 4. When the number of PVA is 1, *b*-ZSM-5 can adsorb on the modified surface. However, due to insufficient modification, ZSM-5 tilts on the modified surface, and ZSM-5 with *b*-orientation cannot form. When the number of PVA molecules is 2, due to the surface having been sufficiently modified with a single-layer structure, ZSM-5 can be stably implanted with *b*-axial orientation on the modified substrate. However, when the number of PVA molecules is 3–5, due to the agglomeration of PVA to form irregularity, ZSM-5 is tilted or embedded in the PVA-modified layer, so that ZSM-5 with *b*-axial orientation cannot form.

**Figure 5:**
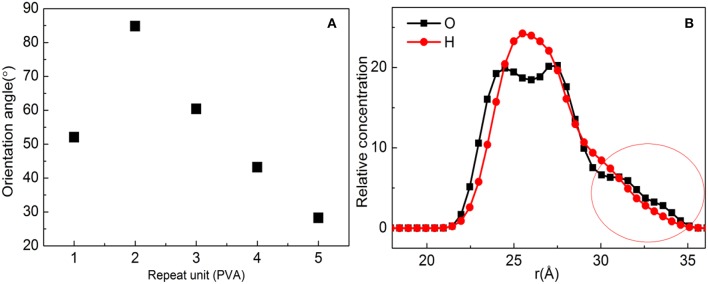
**(A)** Orientation angle between polyvinyl acetate (PVA)/substrate and ZSM-5 with centroid along the *b*-axial direction. **(B)** Concentration profile for H and O atoms with the PVA repeat unit for 2.

In order to further show the orientation degree of ZSM-5 with *b*-axial orientation implanted in the substrate, the orientation angle of the ZSM-5 with substrate is calculated, which is shown in Figure 5A; the orientation angle order with the PVA increasing is 2 > 3 > 1 > 4 > 5, indicating that with the PVA having a molecule number of 2, ZSM-5 has the highest degree of *b*-axis orientation and an orientation angle of 84.81°. However, the theoretical orientation angle is 90°, because the hydroxyl groups of PVA promotes interaction with ZSM-5. Although a uniform layer of modifier formed, the distribution of hydroxyl groups exposed on the surface of the PVA was not uniform (Figure 5B), and the distribution of force with ZSM-5 was uneven, resulting in ZSM-5 not being completely implanted with *b*-axial orientation.

The interaction energies of ZSM-5 and PVA/substrate models can be calculated using

$$\begin{array}{l} {\text{E}}_{\text{inter}}={\text{E}}_{\text{total}}-{\text{E}}_{\text{PVA}/\text{substrate}}-\;{\text{E}}_{\text{ZSM}-5} \end{array}$$

where *E*_total_ is the energy of the ZSM-5/PVA/substrate, *E*_PVA/substrate_ is the energy of the PVA/substrate, and *E*_ZSM−5_ is the energy of ZSM-5. The interaction energies are given in Table 1.

**Table 1:** Interaction energies of ZSM-5/polyvinyl acetate (PVA)/substrate (kcal/mol).

**System** **(PVA unit)**	**None**	**1**	**2**	**3**	**4**	**5**
*E*_inter_ (kcal/mol)	−329.92	−283.12	−447.83	−540.218	−458.352	−633.141

It can be seen from the table that when ZSM-5 is implanted on the surface of the substrate which is not fully modified by PVA, the interaction energy is lower than that of ZSM-5 of the PVA-free-modified substrate. When the surface of the substrate is fully preimplanted with a modifier, the interaction energy is higher than that of ZSM-5 without a PVA-modified substrate. However, it can be seen from the table that the order of interaction energy is 5 > 3 > 4 > 2 > 1 with the increase of the number of PVA repeat units. This is because when the number of PVA molecules exceeds 3 (the thickness is 10 Å), ZSM-5 is embedded in the modifier layer and the contact area with the modifier increases, which leads to the increase of interaction potential. ZSM-5 is stably implanted with *b*-orientation when the number of PVA molecules is 2, and the contact area is small, resulting in low interaction energy.

### *b*-ZSM-5/CTS/Substrate MD Simulation

#### Adsorption of CTS on the Substrate Surface

**Figure 6:**
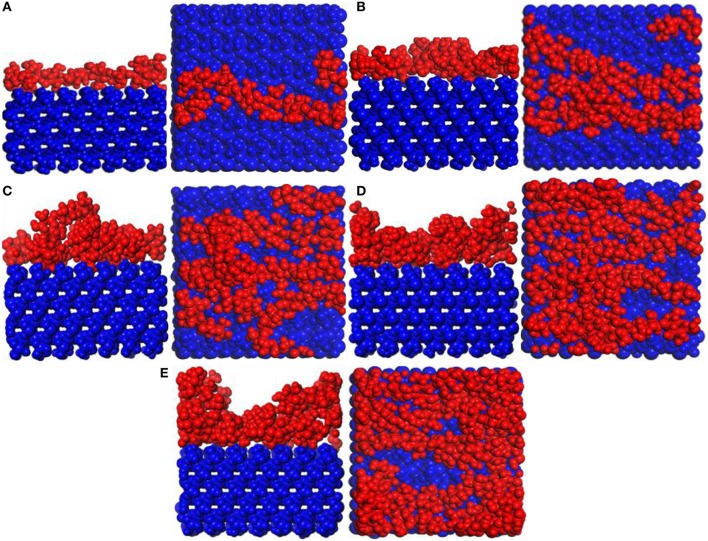
Snapshot diagrams of chitosan (CTS)/α-quartz substrate under molecular dynamics simulation: **(A–E)** the polyvinyl acetate (PVA) repeat units from 1 to 5.

Figure 6 shows snapshot diagrams of the CTS/substrate. Different from the PVA structural unit, the CTS molecular model is linear (Razmimanesh et al., 2015; Asghari et al., 2017), which is hard to agglomerate on the substrate surface. Therefore, it can be seen from Figure 6 that CTS can be stably adsorbed and accumulated on the substrate surface by hydrogen bonding. when the number of molecules is 1–4, the modified layer of CTS on the surface of the substrate is uniform. In contrast, when the number of molecules reaches 5, irregularity formed, because the bulk density of the CTS is too high. It can be also seen that when the number of CTS molecules is 3–4, some atoms in the CTS interface have adhered to the substrate surface.

**Figure 7:**
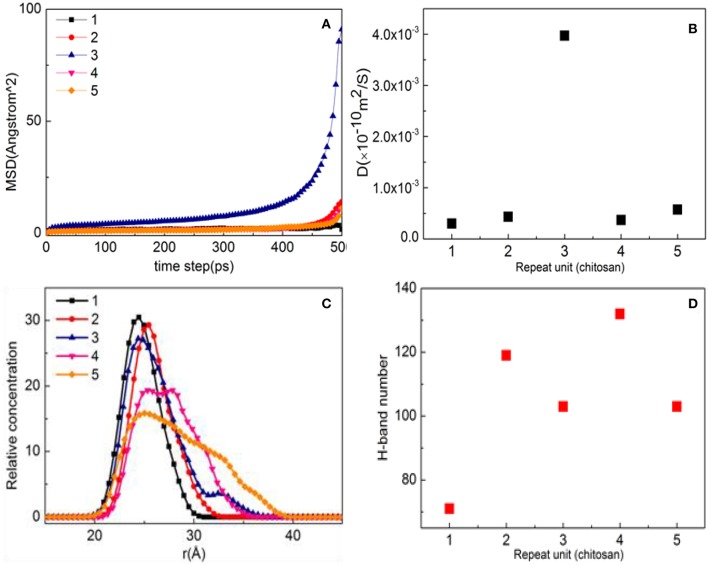
Property analysis of chitosan (CTS)/α-quartz (1 0 0) substrate after equilibrium. **(A)** Mean square displacement (MSD), **(B)** diffusion coefficient, **(C)** hydrogen bond number, and **(D)** relative concentration profile.

As shown in Figure 7A, the MSD with three repeat units is much steeper than that of other molecules, which is hard to preplant on the substrate. When the number of molecules is 1, 2, 4, and 5, the diffusion degree is not intense due to the influence of steric hindrance, indicating that it can be stably adsorbed on the surface of the substrate. When the *D* values in Figure 7B are compared, the order is found to be 3 > 5 > 2 > 4 > 1, indicating that the CTS for a repeat unit of 1 has a strong interaction with substrate, while the repeat unit of 4 comes second.

Figure 7C shows the aggregation morphology of CTS on the substrate surface. When the number of molecules is 1–3, the shape of the peak remains basically the same, and all show a single peak. That is, CTS forms a single modified layer with a thickness of 12 Å. However, when the number of molecules reaches 4, symmetrical peaks are formed at 26.5 and 28.5 Å, indicating that CTS no longer exists as a single modified layer on the surface of the substrate but forms a uniform two-layered layer with a thickness of 20 Å. When the number of molecules is 5, the main peak appears at 26.2 Å and the shoulder peak is formed at 34.4 Å. It can also be seen from Figure 6C that the peak value at 26.2 and 34.4 Å is different, indicating that the modified layer is not uniform. It can be clearly be found from Figure 6D that as the number of molecules increases, the order of hydrogen bond generation is 4 > 2 > 5 ≈ 3 > 1. When the number of molecules is 1–2, the number of hydrogen bonds is small due to insufficient modification. When the number of molecules is 3–4, CTS can fully modify the substrate, so the number of hydrogen bonds formed is the most at the repeat unit of 4 (Figure 7D). However, at the repeat unit of 3, due to the strong degree of CTS movement, the diffusion coefficient is fast, resulting in a decrease in hydrogen bonding, which is consistent with the law of diffusion coefficient illustrated in Figure 7B.

#### Adsorption of b-ZSM-5 on CTS/Substrate Surface

**Figure 8:**
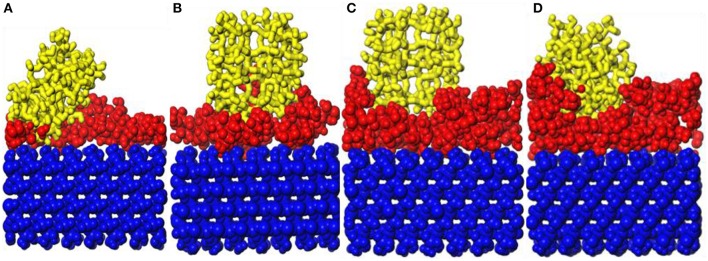
Snapshot diagrams of ZSM-5/chitosan (CTS)/α-quartz under molecular dynamics simulation: **(A–D)** the polyvinyl acetate (PVA) repeat units from 2 to 5.

It can be found in Figure 6 that the surface of the substrate cannot be sufficiently modified at the repeat unit of 1. Therefore, the preplanting study of *b*-ZSM-5 on CTS/substrate is focused on the repeat units of 2–5. It can be seen from Figure 8 that ZSM-5 can adsorb on the surface of the CTS-modified layer. When the number of molecules is 2, surface modification is insufficient, resulting in ZSM-5 inclining on the modified surface. When the number of CTS molecules is 3, the surface of the substrate is sufficiently modified, and a single modified layer of the structure is maintained. Therefore, it can be seen from Figure 8B that ZSM-5 can be stably implanted with *b*-axial orientation. When the number of molecules is 4 (Figure 8C), ZSM-5 can also be implanted with the *b*-orientation due to a double-layered layer. In contrast, when the number of molecules is 5 (Figure 8D), due to the cross-linking of CTS to form irregularity, ZSM-5 is embedded in the CTS-modified layer and cannot form a *b*-axis orientation preplanting. The relative concentration distribution (Figure 7C) displayed that when the number of molecules is 3–4, the thickness of the modifier layer is 12 and 20 Å, so that *b*-ZSM-5 preplanting can be formed either as the thickness of the modifier layer is below 12 Å or beyond 20 Å.

**Figure 9:**
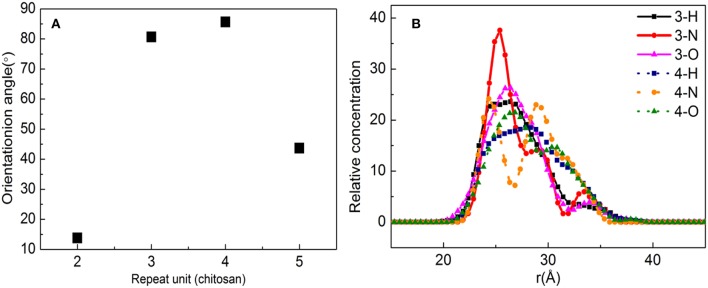
**(A)** Orientation angle between chitosan (CTS)/α-quartz substrate and ZSM-5 with centroid along the *b*-axial direction. **(B)** Concentration profile for H, N, and O atoms with the polyvinyl acetate (PVA) repeat units of 2 and 3.

Figure 9A shows the orientation angle between the *b*-ZSM-5 and the CTS/substrate surface. The orientation angle order is 4 > 3 > 5 > 2, indicating that ZSM-5 has the best *b*-axial orientation and its orientation angle is 85.65° with a molecule number of 4. However, when the number of molecules is 3, the orientation angle is 80.58°, indicating the double-layered modified layer is more suitable for *b*-ZSM-5 preplanting than the single modified layer.

This is because the interacting atoms between CTS and ZSM-5 are H, N, and O. It can be seen from Figure 9B that the concentration distribution of H atoms is relatively uniform, but the concentrations of N and O are quite different. When the number of molecules is 3, due to the strong movement of CTS on the substrate surface, the concentration of N atoms interacting between the CTS layer and substrate is much higher than the concentration of N atoms interacting between the ZSM-5 and CTS layer, indicating that interaction between CTS and ZSM-5 is weak, while when the number of molecules is 4, the peaks of N atoms and O atoms at the molecule number of 3 are much higher than three molecules, and N atoms form a symmetric peak. It is indicated that the main reason that the two-layered modified layer is stronger than the single modified layer is the uniform distribution and concentration distribution of the exposed N and O atoms.

The order of interaction between ZSM-5 and the CTS/substrate surface is as follows: 5 > 3 > 4 > 2; this conclusion can also be obtained by observing their interfacial interaction models after MD simulation (Figures 6, 8). When CTS is insufficiently modified on the substrate (CTS < 2), the interaction energy is less than the interaction energy of ZSM-5 implanted on the surface without the PVA-modified substrate. When CTS is sufficiently modified on the substrate (CTS > 2), the interaction energy is greater than the interaction energy of ZSM-5 implanted on the surface without the PVA-modified substrate. When the number of CTS molecules is five, ZSM-5 is embedded to cause an increase in energy. At the same time, it can be found from Table 2 that the interaction energy of the double modifier (CTS = 4) formed by ZSM-5 and CTS on the surface of the substrate is larger than that of the single modified layer (CTS = 3), which is consistent with the results of Figure 9B analysis.

**Table 2:** Interaction energies of the ZSM-5/CTS/substrate.

**System** **(chitosan unit)**	**None**	**2**	**3**	**4**	**5**
*E*_inter_/kcal/mol	−329.92	−360.01	−484.99	−506.29	−557.38

### *b*-ZSM-5/TiO_2_/Substrate MD Simulation

#### Adsorption of TiO_2_ on Substrate Surface

**Figure 10:**
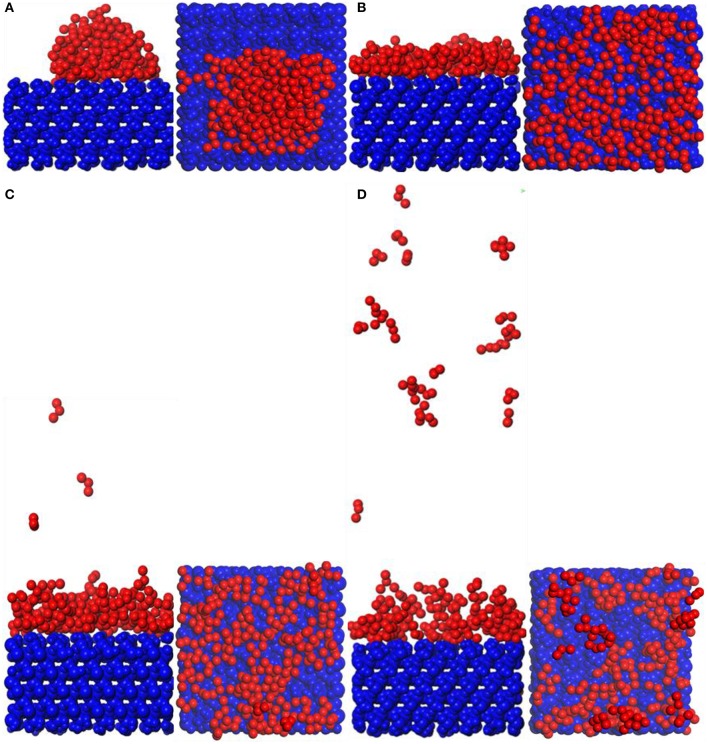
Snapshot diagrams of TiO_2_/α-quartz substrate under molecular dynamics simulation: **(A)** 373, **(B)** 573, **(C)** 873, and **(D)** 1,173 K.

Snapshot diagrams of TiO_2_/substrate is shown in Figure 10. After MD simulation, the TiO_2_ molecules at different temperatures exhibit different formations. At 373–573 K, the adsorption of TiO_2_ on the surface of the substrate belongs to non-dissociative adsorption (Baguer et al., 2009), where the TiO_2_ molecules not only interact with the substrate but also interact with the TiO_2_ molecules to prevent TiO_2_ from separating on the substrate surface to form a dense modified layer. At 873 and 1,173 K, some TiO_2_ molecules are free in the box and cannot be adsorbed on the surface of the substrate, indicating that the adsorption of TiO_2_ belongs to dissociative adsorption. It can also be seen from Figure 10 that because the temperature is low and the kinetic energy necessary for the movement of the TiO_2_ molecule is insufficient at 373 K (Figure 10A), it only shrinks with the formation of the “island” structure on the substrate surface. At 573 K (Figure 10B), TiO_2_ has formed a dense modified monolayer, and the inner TiO_2_ molecules have been embedded in the surface of the substrate. Therefore, Ti–O–Si are easily formed between Ti and Si (SiO_4_ in the substrate surface) (Sang, 2004). At 873 K (Figure 10C), although most of the TiO_2_ is still adsorbed on the surface of the substrate, a small amount of TiO_2_ has been released in the vacuum box, indicating that the kinetic energy is greater than the energy between the TiO_2_ molecules, leading to TiO_2_ dissociation. At 1,173 K (Figure 10D), most of the TiO_2_ molecules have dissociated and the distance between TiO_2_ has become longer, indicating that the kinetic energy is greater than the energy sum for TiO_2_ with substrate and between TiO_2_ molecules. Therefore, the modified layer formed on the surface of the substrate is sparse.

**Figure 11:**
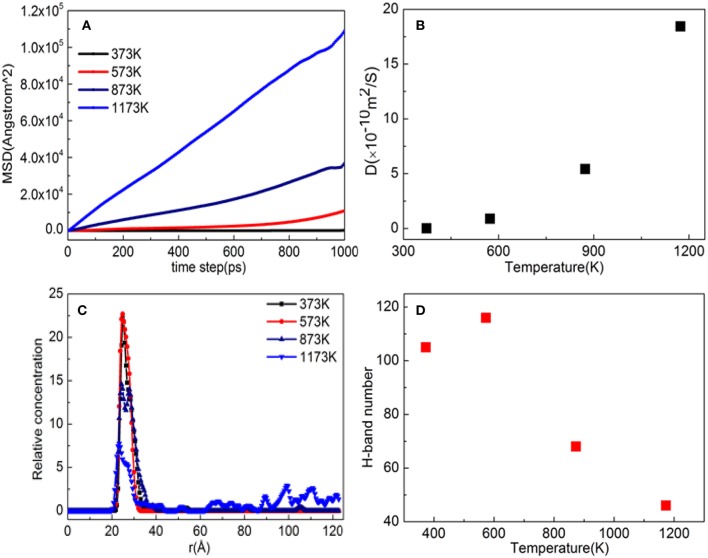
Property analysis of TiO_2_/α-quartz substrate after equilibrium of molecular dynamics simulation. **(A)** Mean square displacement (MSD), **(B)** diffusion coefficient, **(C)** hydrogen bond number, and **(D)** relative concentration profile.

Figure 11A shows the MSD of TiO_2_ on the substrate surface. TiO_2_ as a small molecule is sterically hindered, and the intermolecular interaction is weak, so the MSD curve of TiO_2_ is steep. When the temperature is from 373 to 573 K, the MSD curve of TiO_2_ has a little change, while from 573 to 1173 K, the MSD curve steepness becomes larger. It can also be seen from Figure 11B that the D value is *D*_(373 K)_ ≈ *D*_(573 K)_, *D*_(873 K)_ = 6*D*_(573 K)_, and *D*_(1, 173 K)_ = 21*D*_(573 K)_, indicating that 573 K is the critical temperature for molecules to form non-dissociative adsorption between TiO_2_. When the temperature is higher than 573 K, the kinetic energy of the TiO_2_ molecule increase leads to the destruction of the non-dissociative adsorption and even desorption (Figure 10), resulting in the *D* value increasing drastically. With the increase of temperature, TiO_2_ has undergone the non-dissociation adsorption stage → dissociation adsorption stage → desorption stage on the surface of the substrate. This phenomenon can be visually observed from Figures 11C,D as the temperature increases. The first peak and peak shape are changing. When the temperature is below 573 K, the modified layer still maintains a uniform single modified layer, and the number of hydrogen bonds formed is also increasing, which belongs to a non-dissociation adsorption stage. At 873 K, a symmetrical double peak appeared in the concentration profile, which formed a double modified layer, indicating that the TiO_2_ movement was more intense. Based on the observation of Figure 10D, the number of hydrogen bonds greatly reduced from 573 to 875 K, leading to a great reduction of the interaction between TiO_2_ and the substrate surface, indicating that TiO_2_ is at the dissociative adsorption phase. At 1,173 K, it can be seen from the concentration profile that TiO_2_ peaks appear at 20–120 Å, indicating that hydrogen bonding disappears and that the interaction between TiO_2_ and the surface of the substrate becomes weak. In summary, at 573 K, TiO_2_ is most easily adsorbed on the substrate, and the modified layer maintains uniform flatness.

#### Adsorption of b-ZSM-5 on TiO_2_/Substrate Surface

Through the above MD simulation at different temperatures (Zeng Y. et al., 2015), it is known that TiO_2_ maintains the stage of desorption and dissociation at 1,173 K, so the study for ZSM-5 preplanting on the TiO_2_/substrate surface is focused on 373, 573, and 873 K.

**Figure 12:**
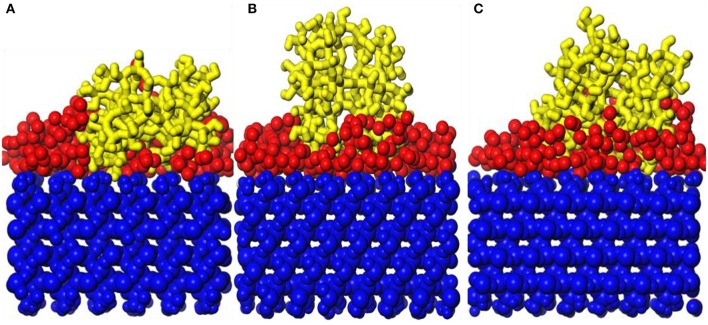
Snapshot diagrams of ZSM-5/TiO_2_/α-quartz under molecular dynamics relaxation: **(A)** 573, **(B)** 873, and **(C)** 1,173 K.

Figure 12 shows snapshot diagrams of the *b*-ZSM-5 on the TiO_2_/substrate surface after MD relaxation. Since the preimplantation temperature of ZSM-5 is 450 K, it can be seen from Figure 12A that the adsorption morphology of TiO_2_ is also changed from the previous island structure to the layer structure. However, when the *b*-ZSM-5 is implanted on TiO_2_/substrate performed under MD simulation at 373 K, the kinetic energy of TiO_2_ is strengthened and the TiO_2_ molecule flows on the substrate surface, so that ZSM-5 was not implanted on its surface at steady state, resulting in the tilt of ZSM-5 on the surface of the substrate (Figure 12A). When *b*-ZSM-5 is implanted on TiO_2_/substrate performed under MD simulation at 573 K, it can be seen from Figure 11B that ZSM-5 can be stably implanted with *b*-axial orientation, and the inner layer of ZSM-5 is embedded in the TiO_2_ modifier layer, indicating that Si–OH (ZSM-5) and Ti are prone to bonding, while when the *b*-ZSM-5 is implanted on TiO_2_/substrate performed under MD simulation at 873 K, it can be seen from Figure 12C that the modified layer has transitioned from the dissociation adsorption stage to the non-dissociation adsorption stage. However, what is similar to the state performed under MD simulation at 373 K is that ZSM-5 tilts on the substrate surface due to the failure to preplant in the steady state. In summary, ZSM-5 is more easily implanted with *b*-axial orientation performed under MD simulation at 573 K.

**Figure 13:**
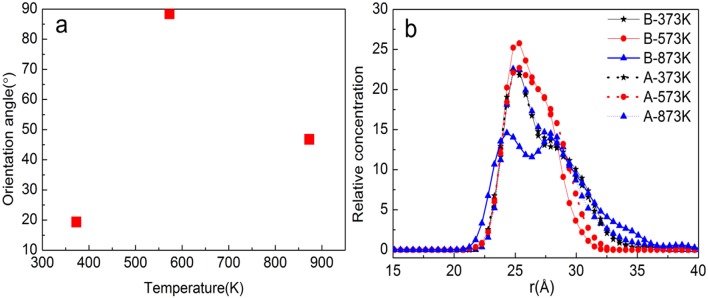
**(a)** Orientation angle between TiO_2_/α-quartz substrate and ZSM-5 with centroid along the *b*-axial direction. **(b)** TiO_2_ concentration profile.

Figures 13A,B show the orientation angle and concentration profile. The order of orientation angle is 573 > 873 > 373 K, indicating that the modifier layer at 573 K is most suitable for *b*-ZSM-5 preplanting, which is 88.42°. Combined with the concentration distribution of TiO_2_ in Figure 13B, the peak of ZSM-5 is high and narrow when *b*-ZSM-5 is implanted under 573 K, indicating that the interaction between ZSM-5 and TiO_2_ results in a denser modification layer. For preplantation at 373 K, as the temperature increased from 373 to 450 K, the kinetic energy of TiO_2_ enhances the movement of TiO_2_, but the concentration and peak shape of TiO_2_ did not change, indicating that the kinetic energy of TiO_2_ was so insufficient at 450 K that part of the “island” structure still exists. Therefore, *b*-ZSM-5 is tilted on the substrate surface. However, as shown in Figure 12B, when the temperature was reduced from 873 to 450 K, the concentration of TiO_2_ after preplanting was much higher than that before preplanting, which was basically the same as that at 573 K, indicating that the adsorption process of TiO_2_ changed from the dissociative adsorption phase to the non-dissociation adsorption phase. However, due to the flow of TiO_2_, ZSM-5 could not be implanted in the steady state, resulting in the tilt of ZSM-5 on the surface of the TiO_2_-modified layer.

Interaction energies of ZSM-5/TiO_2_/substrate are given in Table 3.

**Table 3:** Interaction energies of ZSM-5/TiO_2_/substrate(kcal/mol).

**System** **(TiO_**2**_/temperature)**	**None**	**373**	**573**	**873**
*E*_inter_/kcal/mol	−329.92	−1,119.59	−752.17	−879.61

The order of interaction energy between ZSM-5 and TiO_2_/substrate is 373 > 873 > 573 K. This is because TiO_2_ forms anatase and rutile structures when TiO_2_ is <573 and >873 K, respectively, and the activation property of anatase is higher than that of rutile. The interaction energy of ZSM-5 and TiO_2_/substrate surface preplanting is consistent with that of ZSM-5 in PVA and CTS surface modification and is affected by the orientation degree of *b*-oriented preplanting. The higher the degree of orientation of the *b*-ZSM-5 preplant, the smaller the interaction energy. However, unlike PVA and CTS, since TiO_2_ belongs to a small molecular structure, ZSM-5 is not embedded in the modifier layer, and TiO_2_ easily bonds with Si–O–H on ZSM-5, resulting in much higher energy than that of ZSM-5 implanted on the surface of an unmodified substrate.

### MTA Reaction Test of Materials

**Figure 14:**
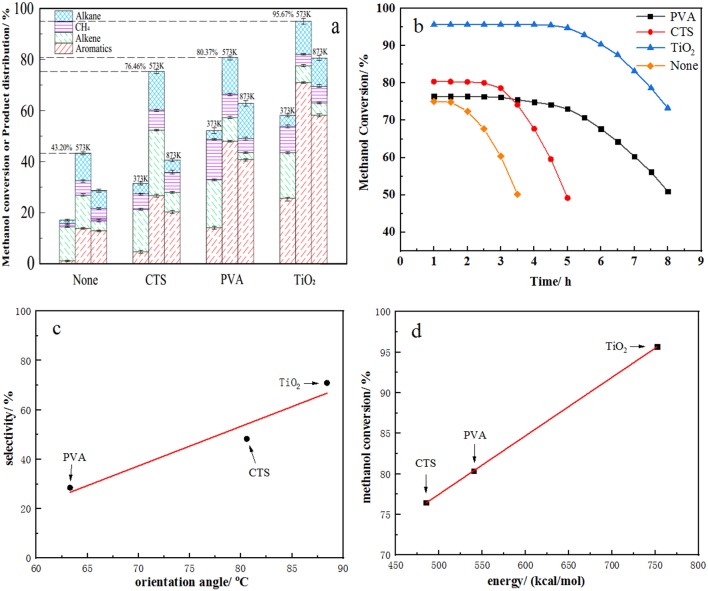
**(a)** Activity and **(b)** lifetime of *b*-oriented ZSM-5 film in methanol-to-aromatics (MTA) reaction. **(c)** Linear fitting curve between orientation angle and selectivity and **(d)** between energy and conversion.

ZSM-5 zeolite catalysts are good catalysts for converting methanol into the hydrocarbons of petroleum-range products. All methanol disappears on HZSM-5 or modified HZSM-5 zeolite in a lot of literature reports. However, the one-pass yield of aromatic hydrocarbons is not necessarily high (Wei et al., 2015; Yang et al., 2017a,b). Surprisingly, in this study, we found that the *b*-ZSM-5/TiO_2_/substrate catalyst produced high benzene, toluene, and xylene (BTX) hydrocarbons (Figure 14A) and exhibited long lifetimes (Figure 14B) in the MTA reaction, although the conversion of methanol decreased slightly. We speculate that this performance of the catalysts can be attributed to two aspects. On the one hand, while in the MTA reaction, methanol is first dehydrated to form dimethyl ether, then the equilibrium product of methanol, dimethyl ether, and water is converted to light olefin, and then light olefins are further reacted to form alkanes and a large number of aromatic hydrocarbons (Dahl and Kolboe, 1994). If the product does not move out of the catalyst surface quickly, the product will react further to form carbon deposit and result in deactivation. In this paper, the dense and uniform *b*-ZSM-5 zeolite films synthesized can provide suitable pore structures and ensure that the residence time of reactants and products in the pore is more conducive to the formation of BTX hydrocarbons. On the other hand, the effective component of ZSM-5 zeolite film supported by quartz carrier is only 5% of that of conventional ZSM-5 solid catalyst at the same catalyst loading volume, but its catalytic activity can still reach 95%, only 5% lower than that of the conventional ZSM-5 solid catalyst. This indicates that the *b*-ZSM-5 zeolite film catalyst can provide more active centers and has better catalytic activity in MTA reaction. Combined with the previous simulation data, we found that the methanol conversion of the catalysts modified by different modifiers correlated with the energy of binding force (Figure 14D) and that BTX selection correlates with the *b*-axis orientation angle of the ZSM-5 film (Figure 14C). Hence, the larger the binding force, the denser the molecular zeolite film is synthesized. In addition, the more appropriate the *b*-axis orientation of the molecular zeolite film, the better the product diffusivity, as the more active sites of olefins are converted into aromatic hydrocarbons. Then the residence time of the aromatic hydrocarbon products is shorter in the catalyst pores, and the aromatic hydrocarbon selectivity becomes higher.

## Conclusion

By employing MD simulation, we have obtained more precise conclusive information of *b*-oriented growth of ZSM-5 crystallites, which highly depends on the surface modifiers. The organic modification mainly forms a modifier layer by cross-linking the hydrogen bond with the substrate, and the flatness of the modified layer is greatly affected by the type and concentration of the modifier. The inorganic modifier not only forms a hydrogen bond and a complex interaction with the substrate but also interacts with others to form a dense layer of modifier, and the degree of tightness of the layer is significantly affected by temperature. PVA is a non-linear organic macromolecule with a large amount of hydroxyl groups on the surface, which easily adheres to the surface of the substrate and agglomerates. When PVA forms a uniform single modified layer of 10-Å thickness, crosslinking takes place, and the modification agent layer is not uniform. CTS is a straight-chain structure with a large amount of hydroxyl and amino groups, which easily accumulate and spread out on the substrate surface, and forms a single modified agent layer of 12-Å thickness and double with 20-Å thickness. TiO_2_ undergoes an island-like (373 K) to layered (573 K) to diffusion-free (>573 K) process on the surface of the substrate, indicating that TiO_2_ undergoes a process of non-dissociative adsorption to dissociation adsorption to diffusion on the surface of the substrate. At 573 K, ZSM-5 is embedded in the modified layer with a *b*-axis orientation, and the binding ability is the strongest. The order of implantation orientation angle of the *b*-axis orientation ZSM-5 on PVA, CTS, and TiO_2_-modified substrate surface is as follows: TiO_2_ (88.42) > CTS (85.65) > PVA (84.81). It shows that ZSM-5 has the best *b*-axis orientation on the surface of the substrate after TiO_2_ modification. In MTA reaction, we found that the *b*-ZSM-5/TiO_2_/substrate catalyst produced high BTX hydrocarbons and exhibited long lifetimes. And the orientation angle is linearly correlated with aromatic hydrocarbon selectivity. Meanwhile, the interaction energy is linearly correlated with methanol conversion.

## Data Availability Statement

The datasets generated for this study are available on request to the corresponding author.

## Author Contributions

RC contributed significantly to the conception of the study. JW performed the data analyses and wrote the whole manuscript. XM and SY helped perform the analysis with constructive discussions. YW contributed reagents, materials, and analysis tools. CS, GZ, and TZ contributed to manuscript preparation.

### Conflict of Interest

The authors declare that the research was conducted in the absence of any commercial or financial relationships that could be construed as a potential conflict of interest.
